# Artemisitene: a promising natural drug candidate with various biological activities needs to confirm the interactional targets

**DOI:** 10.3389/fphar.2023.1221291

**Published:** 2023-06-15

**Authors:** Xian Lin, Jian Chen

**Affiliations:** ^1^ Department of Rheumatism and Immunology, Peking University Shenzhen Hospital, Shenzhen, China; ^2^ Institute of Immunology and Inflammatory Diseases, Shenzhen Peking University-The Hong Kong University of Science and Technology Medical Center, Shenzhen, China; ^3^ Shenzhen Key Laboratory of Inflammatory and Immunology Diseases, Shenzhen, China

**Keywords:** artemisitene, biological activities, rheumatoid arthritis, cancer, inflammatory disease

## Introduction

The road of new drug research and development is full of challenges. To ensure the safety and effectiveness of drugs in patients, new drug research and development are a long screening process of discovering promising compounds and continuously eliminating compounds with obvious side effects. In the history of new drug research and development, natural products are a huge treasure trove and have made great contributions to the treatment of human diseases. For instance, the discovery and research of artemisinin (qinghaosu), an active component extracted from the plant *Artemisia annua* L. helps people defeat malaria. The famous Chinese scientist Youyou Tu shared the 2015 Nobel Prize in Physiology or Medicine for the discovery of artemisinin (Croft and Ward, 2015), which highlights the great development potential of natural products especially traditional Chinese medicine.

Artemisitene (ATT) is a natural compound initially isolated from the herb *Artemisia annua* L. and is an endoperoxide close to the famous antimalaria drug artemisinin (Acton and Klayman, 1985). It was reported that ATT could be obtained from artemisinin through a one-pot selenoxide elimination reaction (Paitayatat et al., 1997), and Avery et al. optimized the synthesis route of ATT (Avery et al., 2003). Over the past few decades, more and more studies show that ATT has a variety of biological activities in multiple human diseases. However, the unclear target protein is one of the most critical bottlenecks affecting the clinical application of ATT. Therefore, it is urgent and necessary to identify the target proteins of ATT in treating multiple human diseases.

## Biological activities of ATT

As an analogue of artemisinin, ATT was shown to have anti-malaria activity (Acton et al., 1993; Paitayatat et al., 1997; Avery et al., 2003). In addition, Chen et al. revealed that ATT serves as a novel nuclear factor erythroid 2-related factor 2 (Nrf2) activator that could induce an antioxidant response in an Nrf2-dependent manner by alleviating Nrf2 ubiquitination and increasing its stability and suppress lung injury induced by bleomycin (Chen et al., 2016). Subsequently, Liu et al. reported that the cysteine (Cys) residues of the cytosolic Nrf2 repressor Kelch-like ECH-associated protein-1(Keap1) might be a potential target contributing to ATT-stimulated Nrf2 activation and is indispensable for stabilizing Nrf2 and facilitating Nrf2-mediated transcription of downstream genes (Liu et al., 2019). Recently, researchers found that ATT could block the production of reactive oxygen species (ROS, especially mitochondrial ROS) and prevent NLRP3 inflammasome assembly and activation, resulting in the decrease of IL-1β production (Hua et al., 2022). Besides, ATT could also suppress NLRC4 and AIM2 inflammasome-mediated IL-1β secretion and IL-6 production, and it has the ability to alleviate ulcerative colitis induced by sulfate sodium salt in mice, suggesting that ATT might regard as a drug candidate in the treatment of inflammatory disorders (Hua et al., 2022). Our recent research also demonstrated that ATT has therapeutic potential for rheumatoid arthritis, an inflammatory autoimmune disease (Chen et al., 2022). In the above study, ATT was shown to have the capacity to manage rheumatoid arthritis by inhibiting proliferation, migration, and invasion, as well as inducing apoptosis of rheumatoid arthritis-fibroblast-like synoviocytes through regulating METTL3/ICAM2/PI3K/AKT/p300 signaling pathway (Chen et al., 2022). It’s worth noting that most reports have focused on the anti-cancer activity of ATT. For instance, in 1993, Woerdenbag et, al. found that the anti-cancer activity of ATT required higher concentrations than the *in vitro* antimalarial activity (Woerdenbag et al., 1993). Years later, Efferth’s group reported that ATT exhibits a cytotoxic effect on multiple cell lines such as cervical carcinoma, leukemia, breast cancer, colon cancer, melanoma, brain cancer, lung cancer, ovarian cancer, and renal cancer, etc., and its anti-cancer activity is superior to some artemisinins (including artemisinin, arteanuine B, arteether, artemether, artesunate) and other compounds exist in *Artemisia annua* L. like scopoletin and 1,8-cineole (Efferth and Oesch, 2004; Efferth et al., 2011). Interestingly, they also demonstrated that ATT regulates iron-related genes to induce ferroptosis, a new form of cell death, contributing to an attractive strategy for cancer treatment (Ooko et al., 2015). Furthermore, our group further revealed that ATT exhibits anti-cancer activity by regulating NEDD4/c-Myc/topoisomerase pathway and inhibits tumor growth in xenotransplanted tumor models (Chen et al., 2018). These findings supported that ATT could be a promising natural drug candidate to treat multiple human diseases.

## Discussion

The target is the junction and pivot of the interaction between pharmaceutical chemical components and the human biological system. The discovery of the target is of great significance to the optimization of the pharmaceutical chemical structure and the interpretation of the effect mechanism. Nowadays, the identification of drug targets is gradually becoming a bottleneck that hinders the development of pharmaceutical chemistry. What’s more, the mechanism of drug action is revealed after their clinical application. For a classic example, artemisinin is used for the treatment of malaria over the past few decades, but its interactional targets are uncertain. It’s exciting that researchers used an alkyne-tagged and biotin-linked artemisinin analogue to identify more than 100 artemisinin covalent binding target proteins, many of which participate in the biological processes essential for the parasite (Wang et al., 2015). This study supported a unifying model to elucidate the action mechanism of artemisinin in killing parasites. However, it is still necessary to further confirm the interactional relationship between artemisinin and target proteins using gold standard methods such as isothermal titration calorimetry, surface plasmon resonance, cellular thermal shift assay, and so on. Previous studies have demonstrated that ATT exhibits excellent biological activities in multiple human diseases and investigating the direct target proteins of ATT became one of the priority issues for promoting the clinical application of ATT. Therefore, it is urgent and necessary to identify the target proteins of ATT in treating multiple human diseases with available drug target screening and validation methods.

In summary, ATT has a variety of biological activities including anti-malaria, anti-anti-cancer, anti-lung injury, anti-ulcerative colitis, anti-rheumatoid arthritis, anti-oxidative stress-related diseases, etc., and could be regarded as a parent compound for structural modification and even a promising natural drug candidate for the treatments of multiple human diseases. Nevertheless, the unclear target protein is one of the most critical limitations affecting the development of ATT. The identification of ATT target proteins will help to study the structure optimization, structure-activity relationship, and molecular mechanism of ATT in treating human diseases, laying a foundation for its clinical application, and also providing a scientific basis for the application of related targets in clinical translational medicine.
